# Increased serum IL-2, IL-4, IL-5 and IL-12p70 levels in AChR subtype generalized myasthenia gravis

**DOI:** 10.1186/s12865-022-00501-8

**Published:** 2022-05-27

**Authors:** Xiao Huan, Rui Zhao, Jie Song, Huahua Zhong, Manqiqige Su, Chong Yan, Ying Wang, Sheng Chen, Zhirui Zhou, Jiahong Lu, Jianying Xi, Sushan Luo, Chongbo Zhao

**Affiliations:** 1 Huashan Rare Disease Center and Department of Neurology, Huashan Hospital Fudan University, No. 12 Middle Wulumuqi Road, Shanghai, 200040 China; 2 National Center for Neurological Disorders, Shanghai, China; 3 Department of Pharmacy, Huashan Hospital Fudan University, Shanghai, China; 4 Department of Neurology, Fujian Medical University Union Hospital, Fuzhou, China; 5 Radiation Oncology Center, Huashan Hospital, Fudan University, Shanghai, China

**Keywords:** Ultrasensitive measurement, Cytokines, Low concentration, Generalized myasthenia gravis, Anti-acetylcholine receptor antibody

## Abstract

**Background:**

Myasthenia gravis (MG) is an autoimmune disorder affecting neuromuscular junctions. Cytokines play important roles in facilitating the immune response and augmenting the pathogenic antibody production. The current study aims to sensitively characterize the serum levels of cytokines with very low concentration in generalized MG (gMG).

**Methods:**

Using ultrasensitive single-molecule arrays (SIMOA), we measured serum IL-2, IL-4, IL-5 and IL-12p70 in 228 participants including 152 immunotherapy-naïve anti-acetylcholine receptor (AChR) subtype gMG from Huashan MG registry and 76 age-matched healthy controls. Subgroup analysis was then performed by stratifying patients according to the onset ages, MGFA classification, disease duration at baseline.

**Results:**

Serum IL-2, IL-4, IL-5 and IL-12p70 levels were significantly elevated in gMG compared to controls (0.179 pg/mL versus 0.011 pg/mL, *P* < 0.0001; 0.029 pg/mL versus 0.018 pg/mL, *P* = 0.0259; 0.215 pg/mL versus 0.143 pg/mL, *P* = 0.0007; 0.132 pg/mL versus 0.118 pg/mL, *P* = 0.0401). Subgroup analysis revealed that IL-2 levels were slightly elevated in gMG with MGFA II compared to MGFA III/IV (0.195 pg/mL versus 0.160 pg/mL, *P* = 0.022), as well as elevated levels of IL-2 (0.220 pg/mL versus 0.159 pg/mL, *P* = 0.0002) and IL-5 (0.251 pg/mL versus 0.181 pg/mL, *P* = 0.004) in late-onset gMG compared with the early-onset gMG. gMG patients with a long duration had a significant increased serum IL-12p70 than those with a short duration (0.163 pg/mL versus 0.120 pg/mL, *P* = 0.011).

**Conclusion:**

Serum IL-2, IL-4, IL-5 and IL-12p70 levels were increased in AChR subtype gMG using ultrasensitive measurement. Serum cytokines with very low concentrations may provide as potential biomarkers in stratifying gMG patients in future prospective cohort studies.

## Background

Myasthenia gravis (MG) is characterized by fluctuating muscle weakness caused by autoantibodies against the acetylcholine receptor (AChR) and the muscle-specific tyrosine kinase (MuSK) [1, 2]. Recent studies identified anti- lipoprotein-related protein 4 (LRP4), anti-Cortactin and anti-Agrin antibodies in serum double-negative MG patients [3, 4]. Approximately, 80% of MG patients develop generalized weakness; 85% of which are seropositive for anti-AChR antibodies [5]. From an immunologic point of view, activated T cells, B cells, and plasma cells, as well as cytokines, play important roles in pathogenic autoantibodies production in MG immunopathogenesis. With the recent advances in technologies, several studies had explored the in-depth peripheral immune profile in generalized MG (gMG) [6, 7]. Cytokine measurement had been greatly facilitated by multiplex assay, which allows multiple analytes to be quantified simultaneously in a single biological matrix sample. With this assay, we had measured 18 inflammatory cytokines in two independent cohorts with myasthenic crisis and non-crisis gMG respectively [8]. However, the sample size for non-crisis gMG was relatively small and we included a certain proportion who have already been on immunotherapies. Second, the detection for cytokines with trace amount is still not satisfactory, which is in consistence with the findings from a normative dataset for plasma cytokines in healthy human adults [9].

Previous studies on MG-relevant inflammatory cytokines mainly focused on Interleukin (IL)-17 (Th17 related), IL-21(Tfh related) and IL-6 (Th17, Tfh, and B cells related) [10–12]. In contrast, some inflammatory cytokines also play key roles in controlling the T helper cell differentiation to effectors and the interplay between T and B cells, such as type 1 cytokines (IL-2, IL-12) and type 2 cytokines (IL-4, IL-5) [13–15]. However, they usually have very low serum concentrations. In the era of rapid development for biologics and small molecular drugs, precise characterization of cytokines in MG remained as an unmet need for monitoring immune dynamics in therapeutic response and future therapeutic investigations. Therefore, our study aims to characterize the serum levels of cytokines with very low concentration in generalized AChR postive MG patients.

Ultra-sensitive single-molecule arrays (SIMOA) provides an alternative and more sensitive method for detecting trace cytokines. [16, 17]. Here we measured IL-2, IL-4, IL-5 and IL-12p70 in the serum from an independent cohort of immunotherapy-naïve gMG patients with positive anti-AChR antibodies using SIMOA. We then performed subgroup analysis within the gMG cohort and correlated the baseline cytokine levels with the short-term clinical outcome.

## Results

### Serum levels of IL-2, IL-4, IL-5 and IL-12p70 in gMG cohort

A total of 228 participants were finally enrolled. Of these, 152 gMG patients (median [IQR] age, 43 [33–60] years; 76 females [50%] and 76 males [50%]) were immunotherapy naïve with positive anti-AChR antibodies and 76 age-matched participants were healthy controls (median [IQR] age, 42 [30–60] years; 38 females [50%] and 38 males [50%]). The median anti-AChR antibodies titer for MG is 5.86 [IQR, 2.47–9.56] nmol/L and thymoma concurrence is 9.9%. We further divided 152 AChR subtype gMG patients into subgroups: (1) Clinical severity according to the Myasthenia Gravis Foundation of America (MGFA): mild group with MGFA II (n = 100) and moderate to severe group with MGFA III/IV (n = 52); (2) Onset ages: early-onset MG (EOMG, onset < 50 years, n = 91) and late-onset MG (LOMG, onset ≥ 50 years, n = 61); (3) Disease duration: short disease duration group (n = 76) and long disease duration group (n = 76). Detailed information regarding each subgroup are listed in Table 1.

**Table 1:** Baseline clinical characteristics in subgroups from immunotherapy-naïve AChR subtype gMG patients

Baseline clinical characteristics	Subgroup 1			Subgroup 2			Subgroup 3		
	MGFA II	MGFA III/IV	*P* sig	Early-onset	Late-onset	*P* sig	Short duration	Long duration	*P* sig
	(n = 100)	(n = 52)		(n = 91)	(n = 61)		(n = 76)	(n = 76)	
Age, median (IQR), years	48.5 (35.3–63)	37 (30–49)	0.005**	35 (29–40)	62.5 (56–66)	< 0.001***	44 (32.5–60.8)	42 (33–59.5)	0.675
Gender, No. (%)			1			0.136			0.105
Male	50 (50)	26 (50)		41 (45.1)	35 (57.4)		43 (56.6)	33 (43.4)	
Female	50 (50)	26 (50)		50 (54.9)	26 (42.6)		33 (43.4)	43 (56.6)	
Osserman Class at first sampling No. (%)			< 0.001***			0.645			0.274
IIa	98 (98)	29 (55.8)		75 (82.4)	52 (85.2)		66 (86.8)	61 (80.3)	
IIb	2 (2)	23 (44.2)		16 (17.6)	9 (14.8)		10 (13.2)	15 (19.7)	
MGFA at first sampling No. (%)			< 0.001***			0.017*			0.322
IIa	46 (46)	0 (0)		22 (24.2)	24 (39.3)		28 (36.8)	18 (23.7)	
IIb	54 (54)	0 (0)		29 (31.8)	25 (41.0)		28 (36.8)	26 (34.2)	
IIIa	0 (0)	25 (48.1)		21 (23.1)	4 (6.6)		9 (11.8)	16 (21.1)	
IIIb	0 (0)	21 (40.4)		13 (14.3)	8 (13.1)		9 (11.8)	12 (15.8)	
IVa	0 (0)	5 (9.6)		5 (5.5)	0 (0)		2 (2.6)	3 (3.9)	
IVb	0 (0)	1 (1.9)		1 (1.1)	0 (0)		0 (0)	1 (1.3)	
AChR-Ab titer, median (IQR), nmol/L	6.2 (2.7–9.5)	5.4 (1.8–10.5)	0.645	4.9 (1.8–9.4)	6.9 (3.5–10.3)	0.089	4.5 (1.9–9.5)	6.4 (2.8–10.3)	0.262
Thymoma, n (%)	8 (8)	7 (13.5)	0.284	8 (8.8)	7 (11.5)	0.587	13 (17.1)	2 (2.6)	0.003**
Disease course, median (IQR), months	5 (2–16.3)	11 (3.3–59.8)	0.008**	7 (3–20)	5 (3–29)	0.577	3 (2–4)	27 (11–61)	< 0.001***

*MG* Myasthenia gravis, *MGFA* Myasthenia Gravis Foundation of America, *AchR* Acetylcholine receptor

*P < 0.05; **P < 0.01; ***P < 0.001

The levels for serum IL-2, IL-4 and IL-5 was higher in immunotherapy-naïve gMG in comparison to HCs (measurable in 98.7% (150/152) versus 82.9% (63/76) for IL-2; 99.3% (151/152) versus 78.9% (60/76) for IL-4; 99.3% (151/152) versus 85.5% (65/76) for IL-5, respectively. For IL-12p70, the levels were equally matched in gMG and HCs (measurable in 83.6% (127/152) versus 84.2% (64/76)).

**Fig. 1:**
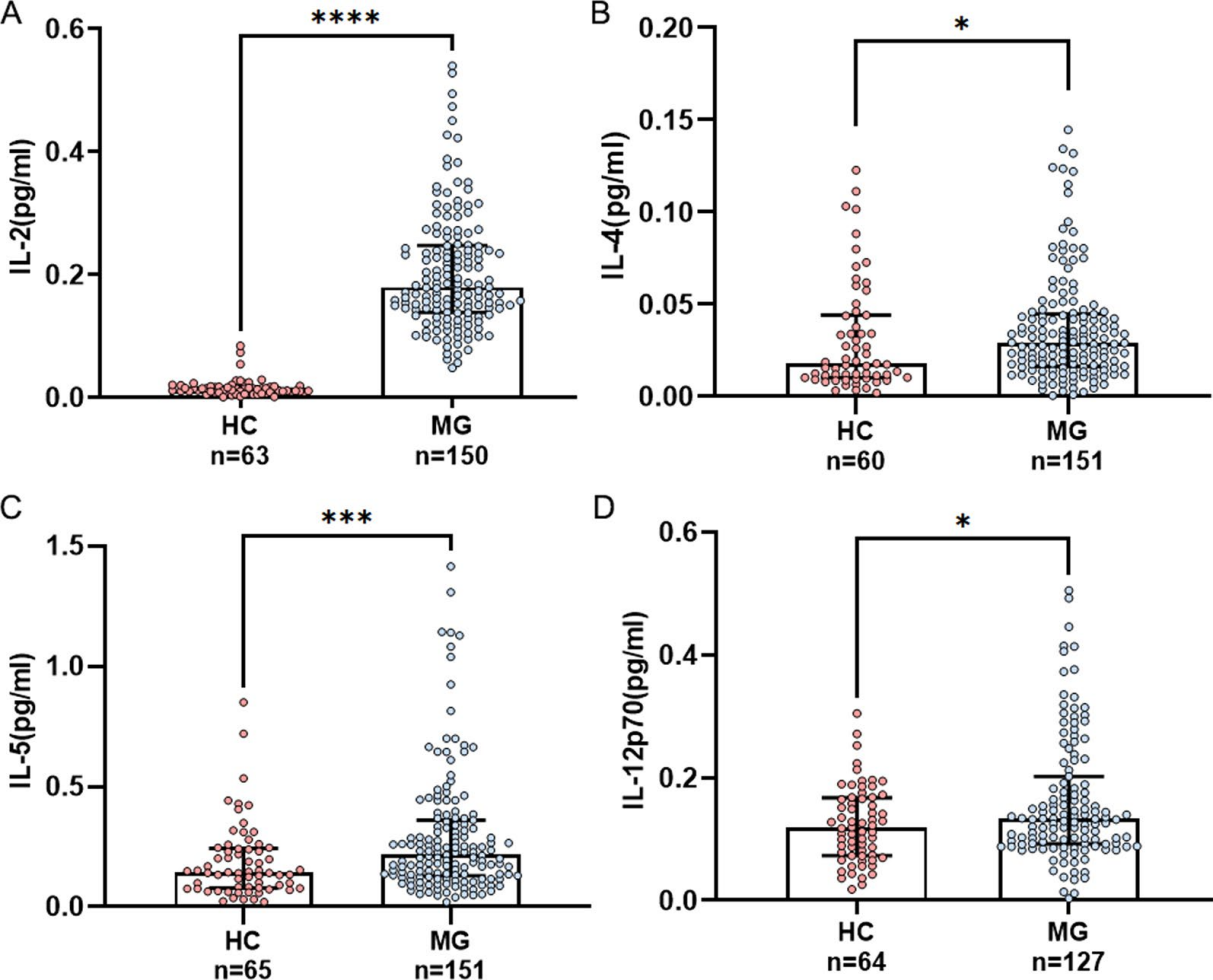
Increased IL-2 (**A**), IL-4 (**B**), IL-5 (**C**), and IL-12p70 (**D**) levels in MG patients compared to the HCs. HC, healthy control; MGFA, Myasthenia Gravis. *P < 0.05, ***P < 0.001 and ****P < 0.0001

Serum IL-2, IL-4, IL-5 and IL-12p70 levels in immunotherapy-naïve gMG patients were significantly higher than those in HCs (median [IQR], 0.179 [0.138–0.247] pg/mL versus 0.011 [0.008–0.017] pg/mL, *P* < 0.0001; 0.029 [0.016–0.045] pg/mL versus 0.018 [0.011–0.044] pg/mL, *P* = 0.0259; 0.215 [0.129–0.360] pg/mL versus 0.143 [0.077–0.243] pg/mL, *P* = 0.0007; 0.132 [0.092–0.202] pg/mL versus 0.118 [0.073–0.167] pg/mL, *P* = 0.0401) (Fig. 1).

### Cytokine measurement in subgroups classified by clinical severity, onset ages and disease duration

**Fig. 2:**
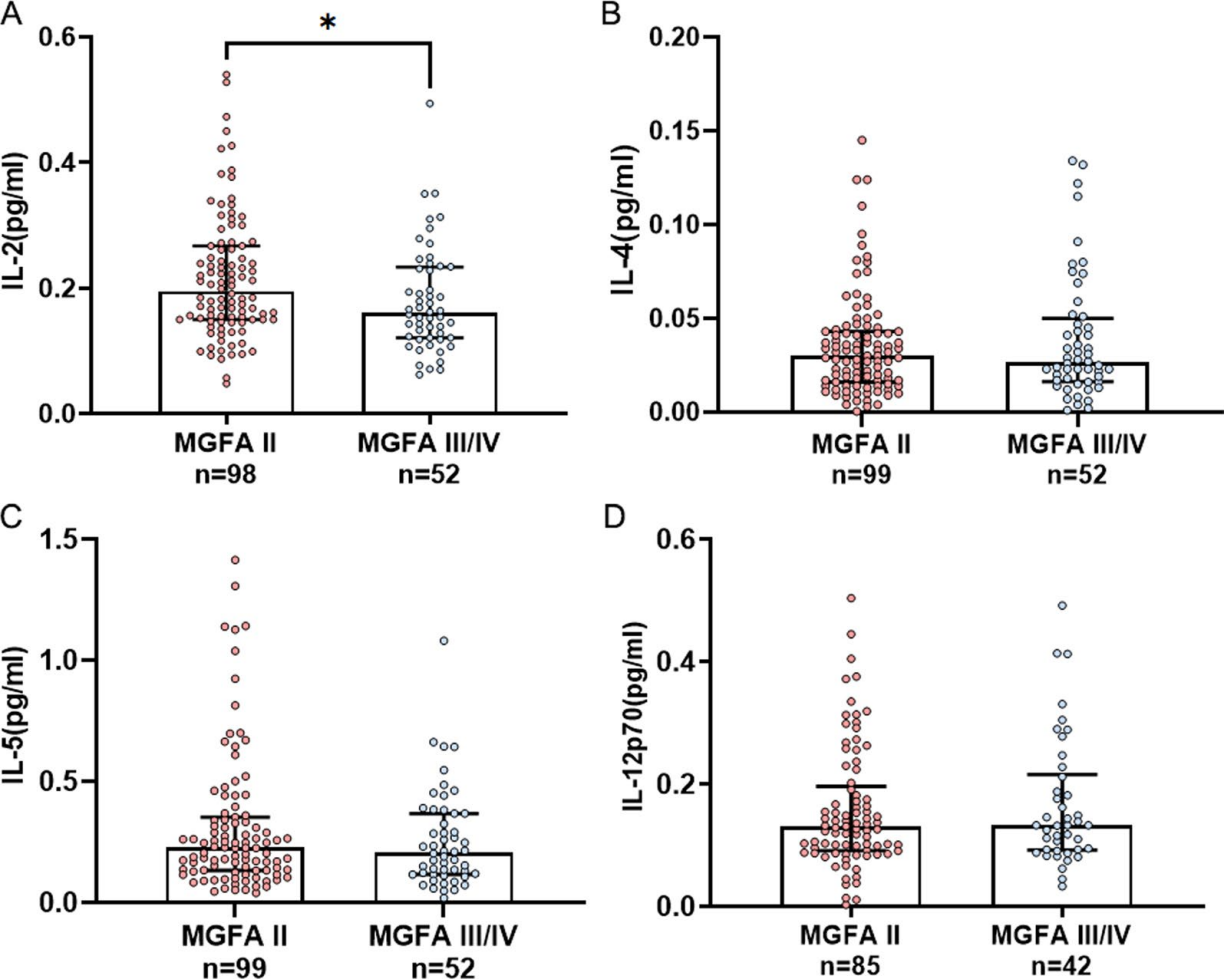
Serum IL-2 (**A**), IL-4 (**B**), IL-5 (**C**), and IL-12p70 (**D**) levels in subgroups stratified by MGFA classifications (gMG with MGFA II versus gMG with MGFA III/IV). MGFA, Myasthenia Gravis Foundation of America. *P < 0.05

Subgroup analysis revealed that gMG patients with MGFA II had increased serum IL-2 compared to those with MGFA III/IV (n = 98, 0.195 [0.150–0.267] pg/mL versus n = 52, 0.160 [0.121–0.233] pg/mL, *P* = 0.022). In contrast, there were no differences in serum IL-4 levels (n = 99, 0.030 [0.016–0.043] pg/mL versus n = 52, 0.026 [0.016–0.050] pg/mL, *P* = 0.931), IL-5 levels (n = 99, 0.226 pg/mL [0.133–0.353] pg/mL versus n = 52, 0.207 [0.117–0.368] pg/mL, *P* = 0.792), IL-12p70 levels (n = 85, 0.131 [0.092–0.197] pg/mL versus n = 42, 0.133 [0.092–0.216] pg/mL, *P* = 0.418) between these two subgroups (Fig. 2).

**Fig. 3:**
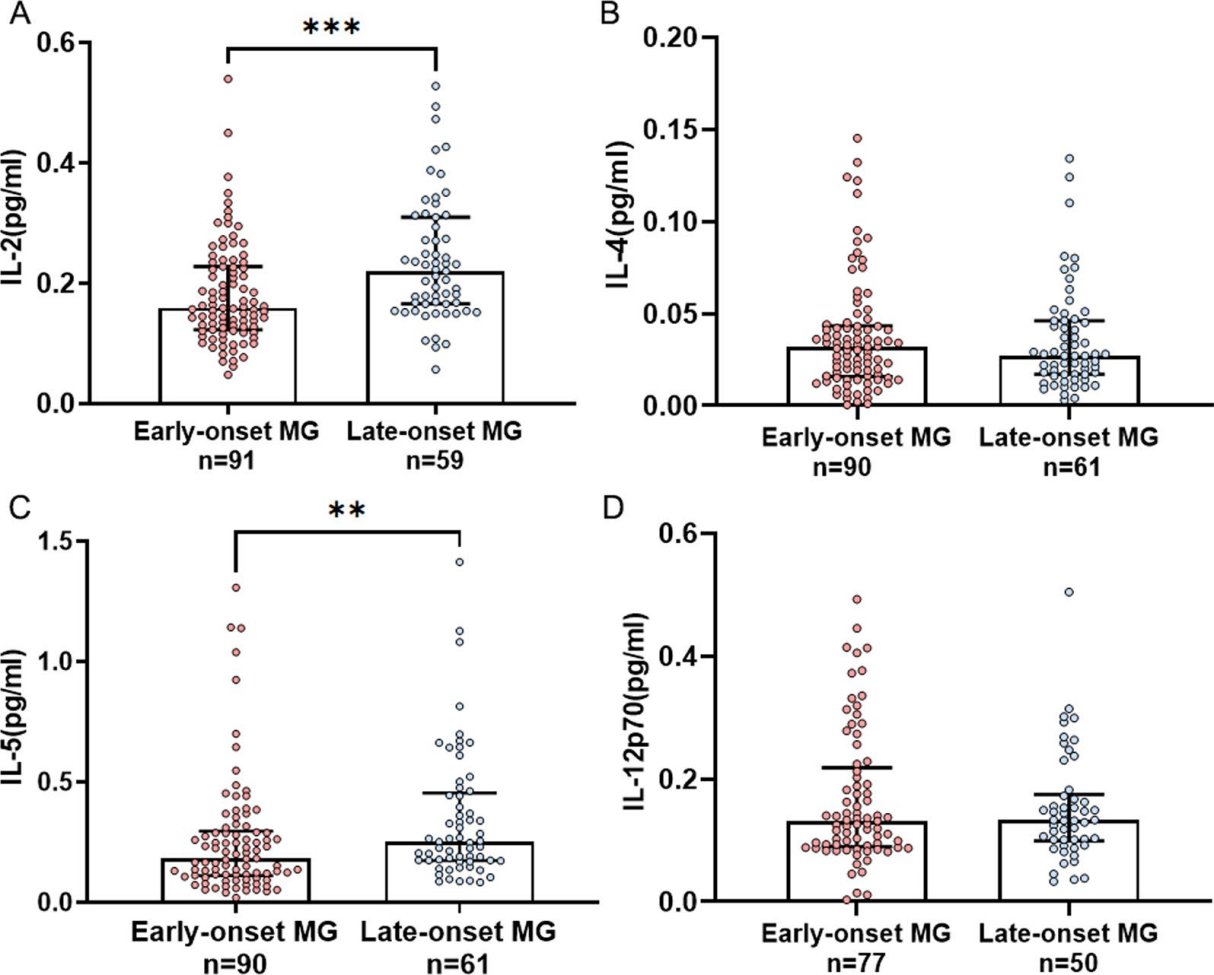
Serum IL-2 (**A**), IL-4 (**B**), IL-5 (**C**) and IL-12p70 (**D**) levels in subgroups stratified by onset age (onset age < 50 years versus onset age ≥ 50 years). *MG* Myasthenia gravis. **P < 0.01, ***P < 0.001

Serum IL-2 was significantly elevated in patients with late-onset gMG in comparison to that in early-onset gMG (n = 59, 0.220 [0.166–0.310] pg/mL versus n = 91, 0.159 [0.123–0.228] pg/mL, *P* = 0.0002). In parallel, serum IL-5 was also elevated in patients with late-onset gMG (n = 61, 0.251 [0.173–0.454] pg/mL versus n = 90, 0.181 [0.111–0.296] pg/mL, *P* = 0.004). In contrast, there were no difference in serum IL-4 nor IL-12p70 levels between these two gMG groups with different onset age (IL-4: n = 61, 0.027 [0.017–0.046] pg/mL versus n = 90, 0.032 [0.016–0.043] pg/mL, *P* = 0.756; IL-12p70: n = 50, 0.133 [0.099–0.175] pg/mL versus n = 77, 0.131 [0.090–0.218]pg/mL, *P* = 0.859) (Fig. 3).

**Fig. 4:**
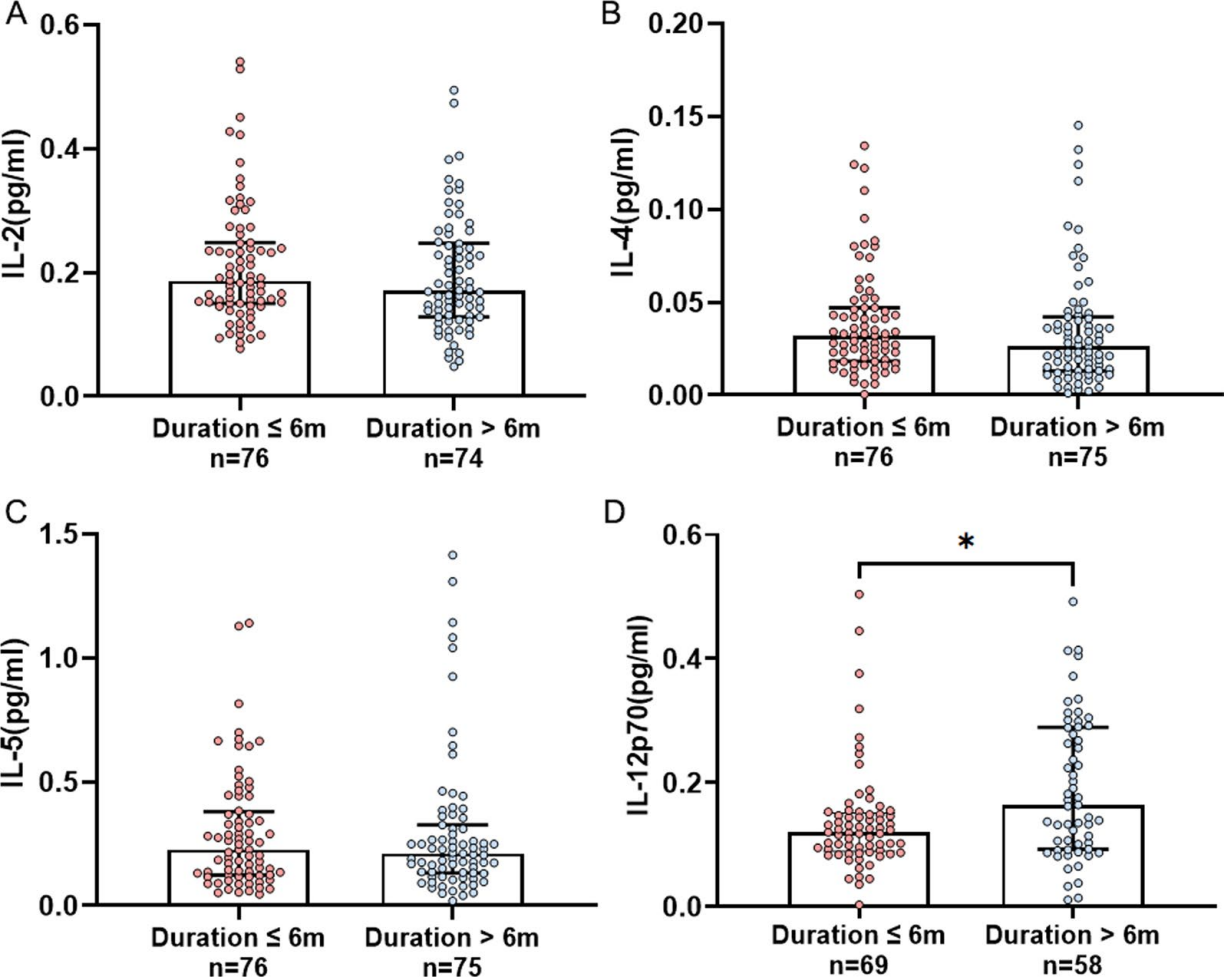
Serum IL-2 (**A**), IL-4 (**B**), IL-5 (**C**) and IL-12p70 (**D**) levels in subgroups stratified by disease duration (duration ≤ 6 months versus duration > 6 months). *P < 0.05

Patients with prior long disease duration had a significant higher elevated serum IL-12p70 level (n = 58, 0.163 [0.093–0.289] pg/mL versus n = 69, 0.120 [0.090–0.151] pg/mL, *P* = 0.011), while no significant differences in IL-2 (n = 74, 0.170 [0.128–0.247] pg/mL versus n = 76, 0.186 [0.150–0.248] pg/mL, *P* = 0.258), IL-4 (n = 75, 0.026 [0.013–0.042] pg/mL versus n = 76, 0.032 [0.018–0.047] pg/mL, *P* = 0.091) and IL-5 (n = 75, 0.210 [0.132–0.325] pg/mL versus n = 76, 0.225 [0.123–0.379] pg/mL, *P* = 0.626) (Fig. 4).

### Correlation of baseline serum cytokines with clinical outcome

We compared the correlation between IL-2, IL-4, IL-5 and IL-12p70 respectively with baseline clinical variables including age, disease course, MG activities of daily living (MG-ADL) score, MGFA-quantitative MG test (MGFA-QMG), MG quality of life 15-item (QoL-15) questionnaire, and MG manual muscle test (MMT). Serum IL-2 had a positive correlation with age (r = 0.306, *P* = 0.0001) and a negative correlation with disease course (r = − 0.172, *P* = 0.036), baseline MG-ADL (r = − 0.166, *P* = 0.0419), QMG (r = − 0.161, *P* = 0.049) and MG-QoL15 (r = − 0.232, *P* = 0.0043). In parallel, serum IL-5 had a positive correlation with age (r = 0.215, *P* = 0.0079) and a negative correlation with QMG (r = − 0.165, *P* = 0.0425). Serum IL-4 was negatively correlated with disease course (r = − 0.164, *P* = 0.046), while IL-12p70 was positively correlated with disease course (r = 0.208, *P* = 0.019). Multivariate analyses indicated that IL-4 levels had positive impact on QMG scores and MMT scores (*P* < 0.05 and *P* < 0.0001, respectively), while cytokines IL-2, IL-5 and IL-12p70 showed no statistically significant difference (Table 2).

**Table 2:** Multiple regression analysis of the effect from serum cytokines on the clinical outcomes

Variables	QMG (1)	MMT (2)	ADL (3)	MG-QoL15 (4)
IL-2 (pg/ml)	− 1.9870 (− 0.53)	− 6.9240 (− 0.85)	− 2.0204 (− 0.84)	− 15.5232 (− 1.52)
IL-4 (pg/ml)	32.8302* (2.51)	78.1857** (2.74)	12.3620 (1.47)	16.0333 (0.45)
IL-5 (pg/ml)	− 2.5695 (− 1.61)	− 2.8166 (− 0.81)	− 0.7753 (− 0.76)	− 3.7432 (− 0.86)
IL-12p70 (pg/ml)	0.2937 (0.07)	1.3330 (0.15)	− 1.5116 (− 0.58)	− 8.1765 (− 0.74)
N	126	126	126	126

*QMG* Quantitative MG test, *MMT* Manual muscle test, *ADL* Activities of daily living, *MG-QoL-15* Quality of life 15-item

*P < 0.05, **P < 0.01; T-statistics are reported in the parentheses

Moreover, the correlations between baseline cytokine levels and the short-term prognosis at 12 months after enrollment were assessed. Baseline IL-12p70 was positively correlated with MG-ADL score at 12 months-follow up (r = 0.253, *P* = 0.025). The other cytokines showed no statistically significant difference.

## Discussion

Many previous studies have addressed the role of cytokines in relation to the pathogenesis of MG. In particular, elevated serum IL-17, IL-6 and IL-21 levels have been demonstrated in anti-AChR antibody-positive MG and are correlated with anti-AChR antibody titer [10–12, 18]. However, some cytokines with very low concentrations remained largely unexplored.

Through the present study, it is confirmed that serum IL-2, IL-4, IL-5 and IL-12p70 levels were increased in AChR subtype gMG using ultrasensitive measurement. In traditional immunological views, IL-2 and IL-12p70 were mainly but not exclusively, produced by polarized Th1 cells. In contrast, IL-4 and IL-5 were produced by polarized Th2 cells and hematopoietic cells [19]. During immune responses, augmented levels of IL-2 and IL-12p70 determine memory potential of antigen specific effector CD8^+^ T cells [20]. IL-4 and IL-5, as type 2 cytokines, were mainly considered to mediate host protective immunity and abundant antibody production [21, 22]. The reciprocal antagonism between Th1 and Th2 cytokines suggests that the immune system needs to suppress inflammatory response which is not required for host protection. Our data also highlighted MG is more complex with prominent combined activities of both type 1 and type 2 cytokines, with multiple cytokines influencing MG progression. In particular, type 2 cytokines (IL-4 and IL-5) may aid in promotion of antibody production from B cells and plasma cells.

Although these cytokines were elevated in current selected cohort, IL-4 and IL-12p70 level were previously revealed to be decreased in non-crisis MG patients compared with healthy controls [8]. We have two explanations for this discrepancy: (1) non-crisis gMG selected in previous study were mainly comprised of MGFA II-IV who had never had a crisis but 25% have already been on immunotherapy, which may lower the peripheral inflammatory cytokines. However, since the measurable levels are relatively low, these findings required more solid evidence; (2) Theoretically, the values detected by SIMOA were more convincing. We reviewed the previous studies employing cross-platforms for immunoassays comparison, the serum levels for cytokines were at different ranges [23–26]. For instance, using healthy controls and MS-derived samples, serum IL-2 levels were reported to be ranged from 0.04 to 0.32 (median:0.09) pg/ml using SIMOA, while the measurement using Milliplex assay were ranged from 0.57 to 16.2 pg/ml (median:1.99) which were 10- to 50-folds higher [23]. Lasseter et al. conducted a comparison analysis on serum derived from patients with post-traumatic stress disorder and Parkinson’s disease and found the lower- and upper-limit of quantification for each cytokine (IL-1β, IL-6, IFN-γ, and TNF-α) were largely varied [24]. The discrepancy can be explained by the following reasons [23, 24, 27]: (1) These immunoassays employed different epitopes for capturing, binding antibodies and signal amplification procedures; (2) Multiplex assays harbor reagent cross-reactivity or undesirable interactions of assay reagents with matrix components and finally measured with the unexpectedly high levels. To be noticed, currently we have no golden standard protocol or method for cytokine measurement. The different immunoassays may yield different results, among which SIMOA and Erenna assays had the highest sensitivity for cytokines detection. However, what remains still unsolved is the “true level” of the cytokines investigated, which can not be largely vary in different assays. More comparisons among ultrasensitive measurements with different techniques are expected to pursue the answer for this question.

The inflammatory profile may vary in clinical settings stratified by the onset age, clinical severity and disease duration for MG patients. The selective and predominant immune responses in different subgroups of gMG patients remained unelucidated. Here we revealed elevated IL-2 levels in mild gMG with MGFA II compared to that with MGFA III/IV. Low-dose IL-2 restores the homeostasis of regular T cells (Treg), which have been demonstrated decreased and impaired in MG patients [28, 29]. Ongoing clinical efforts that capitalize on the early clinical success of IL-2 treatment should bring the use of this cytokine to enhance the biological therapies in autoimmune disorders [30, 31].

Recent clinical studies had emerged regarding the difference in presentations and outcomes between the EOMG and LOMG cohorts. The majority of LOMG had ocular phenotype. The presence of neither anti-titin nor anti-MuSK antibodies points to an unfavorable outcome [32]. Still the diversity in peripheral immune profile between EOMG and LOMG remained unelucidated. A recent study with a novel genomic methodology revealed two T-cell regulators CD28 and CTLA4 which were exclusively linked to LOMG [33]. In vitro studies revealed that inhibition of binding of CTLA-4 to its ligands using soluble anti-CTLA-4 monoclonal antibody during antigen stimuli increased the production not only of IL-2 by Th1 clones, but also that of IL-3 and IFN-gamma by Th1 clones and of IL-3, IL-4, IL-5, and IL-10 by Th2 clones [34]. This is in line with our findings that serum IL-2 and IL-5 levels were significantly elevated in LOMG compared to EOMG, which implicated a T-cell response predomination in the pathogenesis.

Although the concordant elevation in type 1 and type 2 cytokines were demonstrated in gMG, the correlations between baseline cytokines with the short-term clinical outcome revealed some difference. IL-12p70 promotes Th1 immunity thus imposes positive effect in longitudinal autoimmune response for MG. However, the correlations are relatively weak to make any conclusions.

Several limitations in this study are needed to be addressed. First, the normal datasets for all age groups are not available for these cytokines measured by ultrasensitive analysis. Second, the concurrent measurement of peripheral CD4^+^ T lymphocytes and B cells are required to better delineate the role of cytokines in the immunological network. Third, given the current cost and the availability, SIMOA may not be adopted for routine diagnostics in a short time. Ultrasensitive measurement of serum cytokines with very low concentrations in a large longitudinal prospective MG cohort is anticipated in future investigations to monitor the outcome and therapeutic response.

## Conclusion

We confirmed the high levels of serum IL-2, IL-4, IL-5 and IL-12p70 in a cohort of AChR subtype gMG patients. In particular, further analysis revealed significant difference in serum levels of these cytokines in gMG subgroups divided by clinical severity, onset ages and the prior disease duration. These inflammatory serum cytokines may provide as disease biomarkers in stratifying gMG patients in future prospective cohort studies.

### Patients and methods

#### Standard protocol approval, registrations, and patient consents

This study was conducted in accordance with the ethical standards established in the 1964 Declaration of Helsinki and was approved by the Medical Ethics Committee of Huashan Hospital affiliated to Fudan University (2019-441, NCT04535843). Written informed consent was obtained from each participant.

#### Study population

There are 1662 MG patients registered in National center for neurological disorders, huashan hospital, Shanghai from August 8, 2012, through May 31, 2021. MG mimicking diseases including Lambert-Eaton myasthenic syndrome, peripheral neuropathy, myopathies, and motor neuron diseases were excluded before the recruiting. The patients who have already enrolled in previous study for 18 cytokine measurement have been excluded in current study [8]. The inclusion criteria from registration database for cytokine analysis were defined as: (1) onset symptoms and signs compatible with gMG; (2) immunotherapy naïve at baseline; (3) sero-positive for anti-AChR antibody; (4) MGFA clinical classification II to IV; (5) follow-up visits at 12 ± 6 months after baseline recruitment. All eligible participants were aged 18 years or above and were recruited from 17th February, 2017, through 13th March, 2020. Healthy controls (HCs) were enrolled with exclusion of a previous diagnosis of neuroimmune diseases or systemic autoimmune disorders or any fever or sickness at the time of blood sampling.

#### Study design

We retrospectively reviewed the baseline data on age, sex, disease duration, anti-AChR antibody titers, MGFA clinical classification, clinical scales including MG-ADL score, MG QoL-15 questionnaire, MGFA-QMG, and MMT. Subgroup analysis was then performed by stratifying patients by onset ages (early-onset MG versus late-onset MG), MGFA classification (mild group with MGFA II versus moderate to severe group with MGFA III/IV), disease duration at baseline (short duration with less than 6 months versus long duration with longer than 6 months). The MG-ADL score at 12 ± 6 months was also collected as a short-term outcome measurement and correlated with the baseline serum cytokines.

#### Antibody detection

Serum antibodies were measured against AChR using Enzyme-linked immunosorbent assays (ELISA, Euroimmun). The serum from participants were obtained and firstly sent for anti-AChR antibody testing. The optical density was read at 450 nm within 30 min after adding the stop solution. The result was presented as antibody titers using four Parameter-Logistics fitting method to calculate the concentration. The cut-off threshold for anti-AChR antibody is 0.50 nmol/L.

#### Storage and assessment of serum cytokines

Fresh serum was isolated by centrifugation at 3000 × rpm for 15 min at 20 °C, subsequently followed by the preservation at − 80 °C. The selection for these four cytokines was mainly based on: (1) inflammatory cytokines potentially involved in the MG pathogenesis; (2) the cytokines with trace concentrations, which are also poorly investigated in MG cohorts. IL-2, IL-4, IL-5 and IL-12p70 concentrations were finally analyzed in duplicates using SIMOA. IL-2, IL-4, IL-5 and IL-12p70 kits were used with an HD-1 immunoassay analyzer (Quanterix, Boston, Massachusetts, USA). Serum were diluted (IL-4 and IL-5 at 1:2 ratio; IL-2 and IL-12p70 at 1:4 ratio), as recommended by the manufacturer, and the concentrations were calculated using the corresponding standard curve at room temperature. The analyses were performed by a board-certified laboratory technician, blinded to the corresponding clinical data, using one batch of reagents. The intra-assay) for duplicated determinations of concentrations were within 10%, and a CV of lower than 10% was required for an analysis to be considered valid. Inter-assay coefficients of variations were within 12%. More than 80% tests have detectable values for each cytokine.

### Statistical analysis

Continuous variables were presented as median [interquartile range (IQR)] and group comparisons were analyzed using Mann Whitney test as the data were previously tested for not following the normal distribution. Categorical variables were presented as No. (%) and analyzed by Chi-square test. Correlations between serum cytokines and clinical variables of MG cohort were analyzed using Spearman’s method. Multiple linear-regression analyses were then further conducted. We considered a two-tailed adjusted *P* < 0.05 as statistically significant. Data analysis was done using Stata 14.0, and GraphPad Prism 9.0 software. Cytokine concentration with undetectable values were not included in the statistical analysis.

## Data Availability

Data are available upon reasonable request to the corresponding author.

## Abbreviations

MG: Myasthenia gravis
gMG: Generalized MG
AChR: Anti-acetylcholine receptor
MuSK: Muscle-specific tyrosine kinase
LRP4: Lipoprotein-related protein 4
MGFA: Myasthenia Gravis Foundation of America
HCs: Healthy controls
SIMOA: Single-molecule arrays
ELISA: Enzyme-linked immunosorbent assays
QMG: Quantitative MG test
ADL: Activities of daily living
QoL-15: Quality of life questionnaire 15-item
CVs: Coefficients of variations
IQR: Interquartile range
Tfh: T follicular helper
IL: Interleukin
